# Alkali-Activation of Synthetic Aluminosilicate Glass With Basaltic Composition

**DOI:** 10.3389/fchem.2021.715052

**Published:** 2021-08-30

**Authors:** Mohammad I. M. Alzeer, Hoang Nguyen, Christopher Cheeseman, Paivo Kinnunen

**Affiliations:** ^1^ Fibre and Particle Engineering Research Unit, University of Oulu, Oulu, Finland; ^2^ UKCRIC Advanced Infrastructure Materials Laboratory, Department of Civil and Environmental Engineering, Imperial College London, London, United Kingdom

**Keywords:** synthetic silicate glasses, glass reactivity, basalt, alkali activation, AAMs

## Abstract

Alkali-activated materials (AAMs) are a potential alternative to Portland cement because they can have high strength, good durability and low environmental impact. This paper reports on the structural and mechanical characteristics of aluminosilicate glass with basalt-like compositions, as a feedstock for AAMs. The alkali-activation kinetics, microstructure, and mechanical performance of the alkali activated glass were investigated. The results show that AAMs prepared from basalt glass have high compressive strength (reaching up to 90 MPa after 7 days of hydration) compared to those made using granulated blast furnace slag (GBFS). In addition, calorimetry data show that the hydrolysis of the developed glass and subsequent polymerization of the reaction product occur at a faster rate compared to GBFS. Furthermore, the obtained results show that the alkali activation of the developed glass formed sodium aluminosilicate hydrate (N-A-S-H) intermixed with Ca aluminosilicate hydrate gel (C-A-S-H), while the alkali activation of GBFS resulted in predominantly C-A-S-H gel. The developed glass can be formed from carbonate-free and abundant natural resources such as basalt rocks or mixtures of silicate minerals. Therefore, the glass reported herein has high potential as a new feedstock of AAMs.

## Introduction

Increasing demands to reduce green-house gas emissions have driven the development of various new low-carbon binders to replace Portland cement (PC) (Juenger et al., 2011; Naqi and Jang, 2019). PC involves calcination of limestone (CaCO_3_), during which CO_2_ is produced as a by-product. Approximately ∼0.8 tonne of CO_2_ is produced per tonne of PC, and ∼60% of these emissions is from decarbonization of the raw-materials (Stengel and Schießl, 2014). Alkali-activated materials (AAMs) are a potential alternative to PC because they can have high strength, durability and low environmental impact (Provis, 2018). This class of materials can be formed using waste stream raw materials that produce up to 80% less CO_2_ compared to PC (Duxson et al., 2007).

AAMs are usually synthesised using amorphous aluminosilicates that undergo hydrolysis in alkaline media producing 3D polymeric structures (Naqi and Jang, 2019). Typical raw materials for AAMs are clays such as metakaolinite (MK) or industrial by-products such as granulated blast furnace slag (GBFS) and coal fly ash (FA). The chemistry and properties of the alkali-activation reaction products are influenced by the aluminosilicate raw material and the alkali activator (Duxson and Provis, 2008; Juenger et al., 2011). Calcium silicate hydrate (C-S-H) is the main hydration product in PC. In AAMs, aluminum and/or alkali-substituted C-S-H gels are the hydration products. In systems with high Ca content, such as alkali-activation of GBFS, calcium aluminosilicate hydrate gel (C-A-S-H) is the dominant hydration product and this has a tobermorite-like structure, with Si present mostly as Q^2^ with some Q^1^ and Q^3^ units (Provis et al., 2015; Gomez-Zamorano et al., 2017). In the case of Ca-deficient systems, such as alkali activation of MK or FA, sodium aluminosilicate hydrate gel (N-A-S-H) or sodium aluminum substituted calcium silicate hydrate (C-(N)-A-S-H) are the main hydration products. These contain a highly crosslinked 3D structure (Si present in mainly Q^4^ units) (Provis et al., 2015; Gomez-Zamorano et al., 2017). The gels phases can co-exist and this depends on the composition of the raw materials or the presence of both high and low Ca content raw materials (Bernal et al., 2011; Ismail et al., 2014). The raw materials typically used for AAMs are in high demand as supplementary cementitious materials (SCMs) for use in the concrete industry (Provis and Bernal, 2014; Provis, 2018). In addition, the availability and reserves of such materials are limited. Recent developments using sustainable energy sources has resulted in coal-fired power plants being phased out in many countries, and as a result the supply of coal FA is limited. Similarly, steel recycling as well as the move towards direct reduction of iron has limited the supply of GBFS (McCarthy et al., 2017). The search for alternative raw materials that could be used in the synthesis of AAMs is therefore of high significance.

Various industrial wastes have been suggested for this purpose and these include Fe-rich clays (McIntosh et al., 2015), red mud (Hertel and Pontikes, 2020; Hoang et al., 2020), ground coal bottom ash (Sathonsaowaphak et al., 2009; Topçu et al., 2014; Lancellotti et al., 2015), biomass ash (Rajamma et al., 2012; Natali Murri et al., 2015; Shearer et al., 2016; Font et al., 2020). In addition, post-consumer ground waste glass has also been studied in the preparation of AAMs (Martinez-Lopez and Ivan Escalante-Garcia, 2016; Wang et al., 2016; Torres-Carrasco and Puertas, 2017; Lu and Poon, 2018; Liu et al., 2019). However, the use of ground waste glass as a sole precursor fails to produce AAMs with the desired mechanical properties (Liu et al., 2019). The various contents of oxides in waste glass result in inconsistent strength of AAMs, even when they are applied as fine aggregates in AAMs mortars. The Ca and Al content in waste glass is typically very low, therefore the main hydration product of alkali activated waste glass is Si-high, Ca and Al-low gel (Torres-Carrasco and Puertas, 2017) which has different macrostructural properties compared to precursors with high Ca and Al content. The reuse of waste glass in the synthesis of AAMs has been well reviewed elsewhere (Liu et al., 2019).

Alkali activation of synthetic glasses has also attracted much attention recently (Kinnunen et al., 2019; Nie et al., 2020). Materials such as coal FA and GBFS are mainly composed of a glassy phase (with up to 60 and 90 wt% of glass in FA and GBFS respectively) (Douglas and Zerbino, 1986; Ward and French, 2006). This glassy phase is responsible for the pozzolanic and/or hydraulic reactivity (Schöler et al., 2017; Kucharczyk et al., 2019). The reactivity of silicate glasses is generally proportional to the degree of depolymerization of the glass network. The degree of network depolymerisation increases with the content of alkali or alkali earth cations (known as network modifiers) (Schöler et al., 2017; Kucharczyk et al., 2019). The presence of network modifiers disrupts the internal order within the silicon tetrahedral units by breaking the Si-O-Si bonds which increases the number of non-bridging oxygens and increases the degree of depolymerisation (Nie et al., 2020).

Compared to other industrial and agricultural wastes, synthetic glasses demonstrate high potential as raw material for cementitious applications because they can be synthesized with tailored compositions and characteristics. Several studies have recently reported the alkali activation of synthetic glasses with composition similar to the glassy phases present in industrial wastes, such as BFS and FA, for the aim of correlating their structural characteristics and reactivity (Moesgaard et al., 2010; Durdziński et al., 2015a, 2015b; Schöler et al., 2017; Kucharczyk et al., 2019; Nie et al., 2020). However, the potential of synthetic glasses as a new source for AAMs has rarely been investigated.

This paper reports on the structural and mechanical characteristics of synthetic aluminosilicate glass derived from basalt compositions (designated as BG) as a feedstock for AAMs. The glass composition and its structural characteristics have recently been reported by (Kinnunen et al., 2019). Although the use of basalt fibres as reinforcement in cement and geopolymers have been investigated (Timakul et al., 2016; Punurai et al., 2018), the use of basalt as a raw material for AAMs has not been reported. Here the alkali-activation of BG in terms of reaction kinetics, phase assemblage, microstructure, and mechanical properties were investigated. The results were compared with AAMs prepared from other common raw materials such as GBFS.

In the current study, some of the used raw materials in the glass synthesis are in the carbonate form (e.g. CaCO_3_, MgCO_3_, and Na_2_CO_3_) which generates CO_2_ during melting, and thus does not guarantee low total CO_2_ emissions. However, the synthesised glass could be obtained from naturally occurring carbonate free silicate minerals as the sole raw materials. For instance, similar chemical composition could be obtained by melting a mix of anorthite (CaAl_2_Si_2_O_8_), olivine ((Mg, Fe)_2_SiO_4_), wollastonite (CaSiO_3_), and Na_2_O (Kinnunen et al., 2019). In addition, the chemical composition of the synthesised glass is similar to basalt, and thus it could be obtained from basalt rocks or basalt rock dust. Hence, the glass presented herein could be synthesised with low raw materials-related CO_2_ emissions. The PC production, on the other hand, produces ∼0.8 kg of CO_2_ for each kg of cement (Stengel and Schießl, 2014). Regarding carbon emissions due to energy consumption, it is estimated that the energy consumption for PC production is about 3.7 GJ/ton clinker. In this regard, the energy consumption of glass production is comparable to that of PC clinker in which a melting tank operated at 1,600 C consumes ∼3.8 GJ/ton (Sardeshpande et al., 2007; Kinnunen et al., 2019). Therefore, the present work provides a potential new raw material for AAMs with low carbon footprint.

## Experimental

### Glass Preparation

The glass was prepared following the procedure described elsewhere (Kinnunen et al., 2019). In a typical batch, an amount of SiO_2_ (99% metals basis, Alfa Aesar), Al_2_O_3_ (activated neutral, Sigma Aldrich), Na_2_CO_3_ (BioUltra, anhydrous ≥99.5%, Sigma Aldrich), CaCO_3_ (VWR Chemicals), MgCO_3_ (basic, extra pure, ACROS ORGANICS), Fe_2_O_3_ (99.9% metal basis, Alfa Aesar), and TiO_2_ (anatase, 99.7% metals basis, Sigma Aldrich) were mixed manually and homogenised by milling at 1,200 rpm for 1 min using a rotary disc mill (Retsch RS 200).

The mix was melted in a Pt crucible using a Nabertherm high temperature furnace. The powder batch was heated to 1,050 C for 2 h (at 20 C.min^−1^) and then the temperature was increased to 1,600 C (at 10 C.min^−1^) and held for 2 h. The melt was cast onto a water-cooled Cu plate and the glass formed was ground in a rotary disc mill (Retsch RS 200) operated at 1,400 rpm for 1 min. The powder used in subsequent experiments was obtained by sieving through a 44 µm sieve.

### Alkali Activation

10 M NaOH (98.7%, VWR chemicals) solution was used as the activating solution and the specimens were prepared with a liquid: solid ratio of 0.4 following the procedure reported elsewhere (Sun and Vollpracht, 2018; Sun et al., 2020). The mix was stirred manually for 3 min followed by stirring at 1,000 rpm for another 5 min using a laboratory electric mixer. The pastes formed were poured into rectangular 10 × 10 × 40 mm molds, sealed, and cured at 40 C for 24 h. The samples were then cured at room temperature until tested. The performance of the alkali activated basaltic glass samples (designated as BG-AA) were compared with pastes prepared from granulated blast furnace slag (KJ400, obtained from SSAB Raahe, Finland), designated as GBFS-AA. The paste formed using GBFS was prepared in the same manner as BG-AA samples using 10 M NaOH as the activator with 0.4 liquid: solid ratio.

**FIGURE 1:**
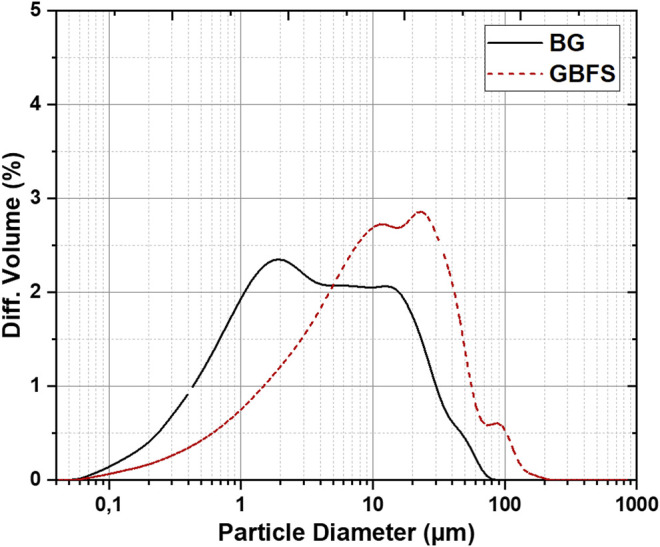
Particle size distribution of the materials used in this study.

The chemical composition and particle size distribution (PSD) of the synthesized glass and GBFS used are given in Table 1 and Figure 1. The molar ratios of the prepared AAMs are shown in Table 2.

**TABLE 1:** Chemical composition (wt% as measured by XRF), and the particle size distribution of the materials used in this study.

Oxide	BFS	BG
SiO_2_	34.3	39.8
Al_2_O_3_	9.1	20.5
CaO	37.9	18.6
MgO	9.8	8.7
Na_2_O	0.6	5.4
Fe_2_O_3_	0.8	5.5
TiO_2_	1.4	1.5
SO_3_	2.8	—
K_2_O	0.6	—
MnO	0.3	—
SrO	—	—
BaO	—	—
P_2_O_5_	—	—
Total	97.6	100.0
D50^a^	10.3	3.3

a Median particle size (µm).

**TABLE 2:** Chemical composition of the synthesised AAMs.

Molar ratio	BG-AA	GBFS-AA
SiO_2_/Al_2_O_3_	3.30	6.35
Na_2_O/SiO_2_	0.43	0.37
H_2_O/SiO_2_	3.35	3.92
CaO/SiO_2_	0.50	1.20
MgO/SiO_2_	0.33	0.43

### Characterisation

The PSD was determined by using a Beckman Coulter LS 13 320 laser diffraction particle size analyser. The heat of hydration of pastes was studied using isothermal calorimeter (TAM Air) at 40 C over a 7-day period. 10 M NaOH solution was used as the reference sample. The pastes were mixed *ex-situ* for 1 min, with a liquid: solid ratio of 0.4, using a laboratory shaker (Vortex-Genie 2). The phase assemblage and microstructural analysis of alkali activated materials were conducted on samples after 7 days hydration. Thermogravimetric analysis (TGA, Precisa PrepASH 129 Thermogravimetric Analyzer) used approximately 0.5 g of sample heated from room temperature to 1,000°C, at a heating rate of 10 ^°^C/min, under a N_2_ atmosphere.

XRD patterns were obtained using a X’Pert PRO X-ray diffractometer with Cu Kα1 radiation operated at 45 kV and 40 mA. The phase identification was obtained using X’pert HighScore Plus (Malvern PANalytical software). DRIFT FTIR spectra in the range of 500–4,000 cm^−1^ were collected on powder samples using a Bruker Vertex v80 spectrometer.

The microstructure of paste samples was observed using scanning electron microscopy [SEM, Zeiss Ultra Plus (Germany)] with a 15 kV accelerating voltage, with backscattered electrons (BSE) at a working distance of 8–9 mm. Samples were collected after mechanical testing and hydration inhibited by solvent exchange using isopropanol. The samples were then cast into low-viscosity epoxy resin by vacuum-impregnating. After the hardening of the resin, samples were polished with diamond discs (to 1-μm size) at 150 rpm using ethanol as lubricant. The samples were carbon coated (Vacuum Evaporator JEE-420T, JEOL, Japan). Chemical compositions were determined using an X-Max EDS detector (Oxford Instrument, the UK), while EDS hypermaps were acquired using Oxford Instrument Aztec software. The maps were set to a resolution of 1,024 × 768 pixels and a dwell time of 200 μs for a total 10 frames. These were then quantified using the QuantMap function in the Aztec software for all elements without any normalization. These maps were used as inputs for further EDS analyses using the edxia method (Georget et al., 2021) in Glue’s interface (Robitaille et al., 2017).

XRF analysis was conducted using AxiosmAX X-ray fluorescence spectrometer. The pastes were cast in 10 × 10 × 40 mm molds and the compressive strength after 1 and 7 days of hydration was measured using Zwick/Roell Z010. After 28 days of hydration, the compressive strength was measured by Zwick/Roell Z100 machine with a force rate of 2.4 kN/min following BS EN 196–1 standard. The measurement was done in triplicate, and the results are expressed with the standard error (standard error = standard deviation/√n; where n is the number of samples).

## Results

### Isothermal Calorimetry

**FIGURE 2:**
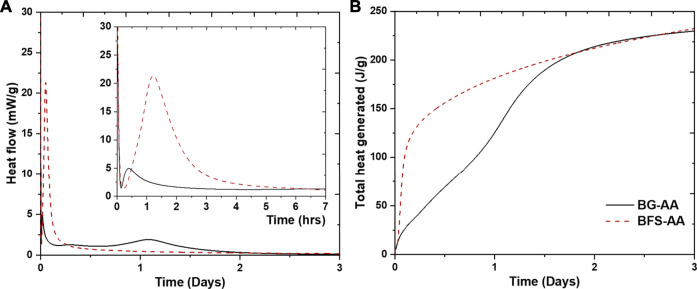
Isothermal calorimetry data for the studied pastes obtained at 40°C **(A)** normalised heat flow (mW/g), and **(B)** cumulative heat generated (J/g).

Figure 2 shows the isothermal calorimetry data for the prepared AAMs. Alkali activated basaltic glass (BG-AA) displays three exothermic peaks while only two were recorded for the alkali activated granulated blast furnace slag (GBFS-AA). The initial exothermic dissolution peak, typically appears once the alkali activator is added to the solid, could not be recorded because the samples were mixed *ex-situ* as described earlier. The decline of this initial peak can be seen in Figure 2A. The second exothermic peak appears within the first 3 h of the alkali reaction for all the alkali activated materials. This peak is associated with the first sharp increase in the total heat generated (Figure 2B). The third peak appears after 1 day of hydration for the BG-AA (Figure 2A) and causes the second rise of the total heat generated (Figure 2B).

### XRD

**FIGURE 3:**
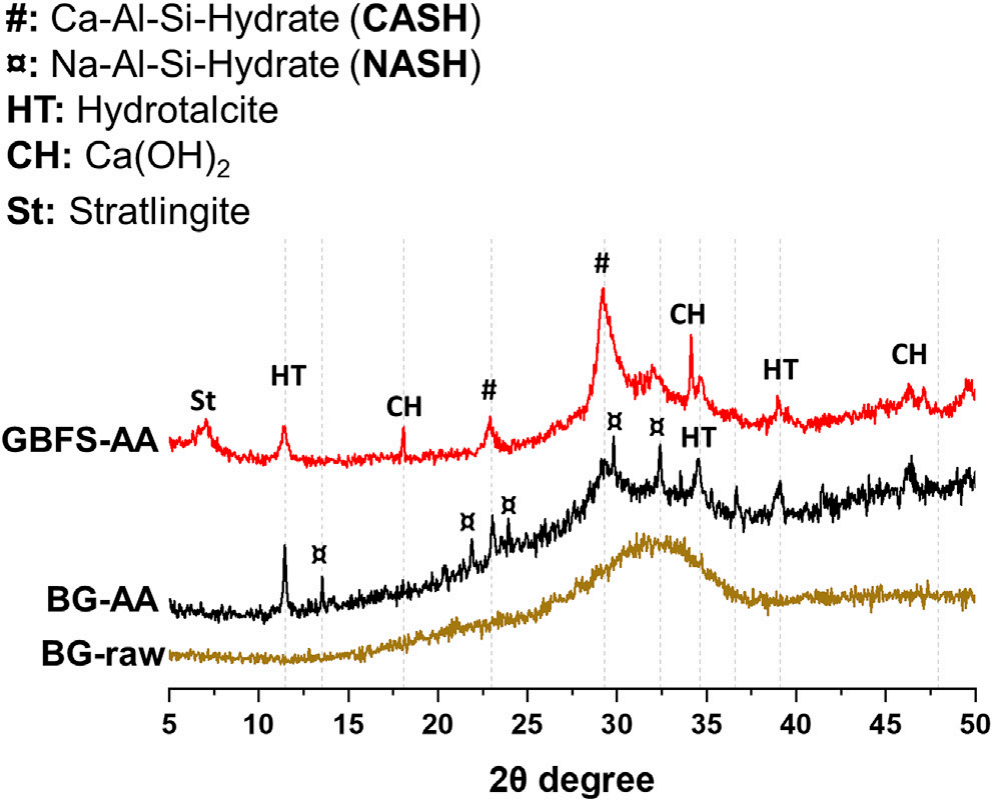
XRD patterns for the raw BG and the alkali activated materials.

The XRD patterns for the raw BG and the synthesised AAMs are presented in Figure 3. The raw glass displays broad amorphous background in the range of 25–40 2θ° with no crystalline phases detected. The BG-AA shows the formation of various hydrated phases mainly sodium aluminosilicate hydrate (N-A-S-H), hydrotalcite (Mg_6_Al_2_(OH)_16_CO_3_.4H_2_O), and calcium aluminium silicate hydrate (C-A-S-H). In the case of GBFS-AA, C-A-S-H, hydrotalcite, portlandite (Ca(OH)_2_) and stratlingite (Ca_4_Al_2_(OH)_12_ [AlSi(OH)_8_]_2_.2H_2_O) were identified as the main hydration products.

### TGA/DTG

**FIGURE 4:**
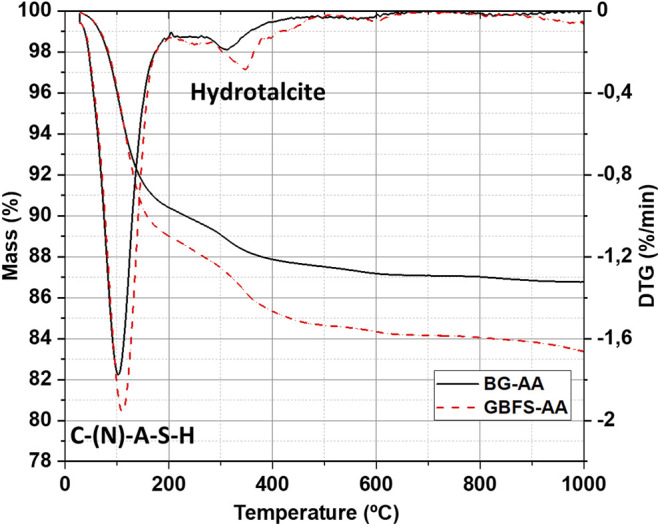
TGA and DTG profiles for the prepared alkali activated materials.

The formation of the reaction products was also studied by TGA/DTG as presented in Figure 4. The TGA profiles for the AAMs one major weight loss in the range of 50 and 200 °C. Another weight loss in the range of 200 and 400 °C only appears in the alkali activated BG and GBFS.

### FTIR

**FIGURE 5:**
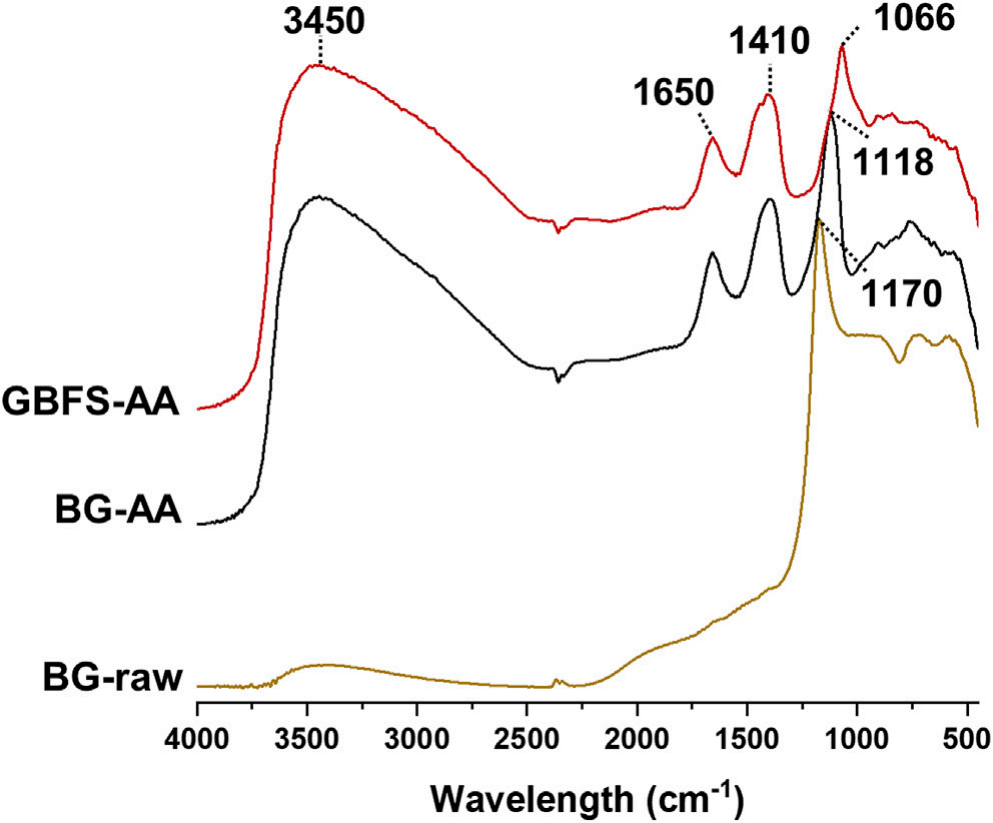
DRIFT FTIR spectra for the raw BG and the alkali activated materials.

Figure 5 shows the DRIFT FTIR spectra of the raw BG glass as well as the prepared AAMs. The AAMs displays 4 main peaks: one major peak at ∼1,100 cm^−1^ corresponding to Si-O asymmetric stretching vibration. another two peaks at ∼3,450 and 1,650 cm^−1^ which are assigned to the stretching and bending modes of H-O respectively. The fourth peak at ∼ 1,410 cm^−1^ is associated with stretching vibration of C-O in carbonates species due to atmospheric carbonation.

### SEM/EDS

**FIGURE 6:**
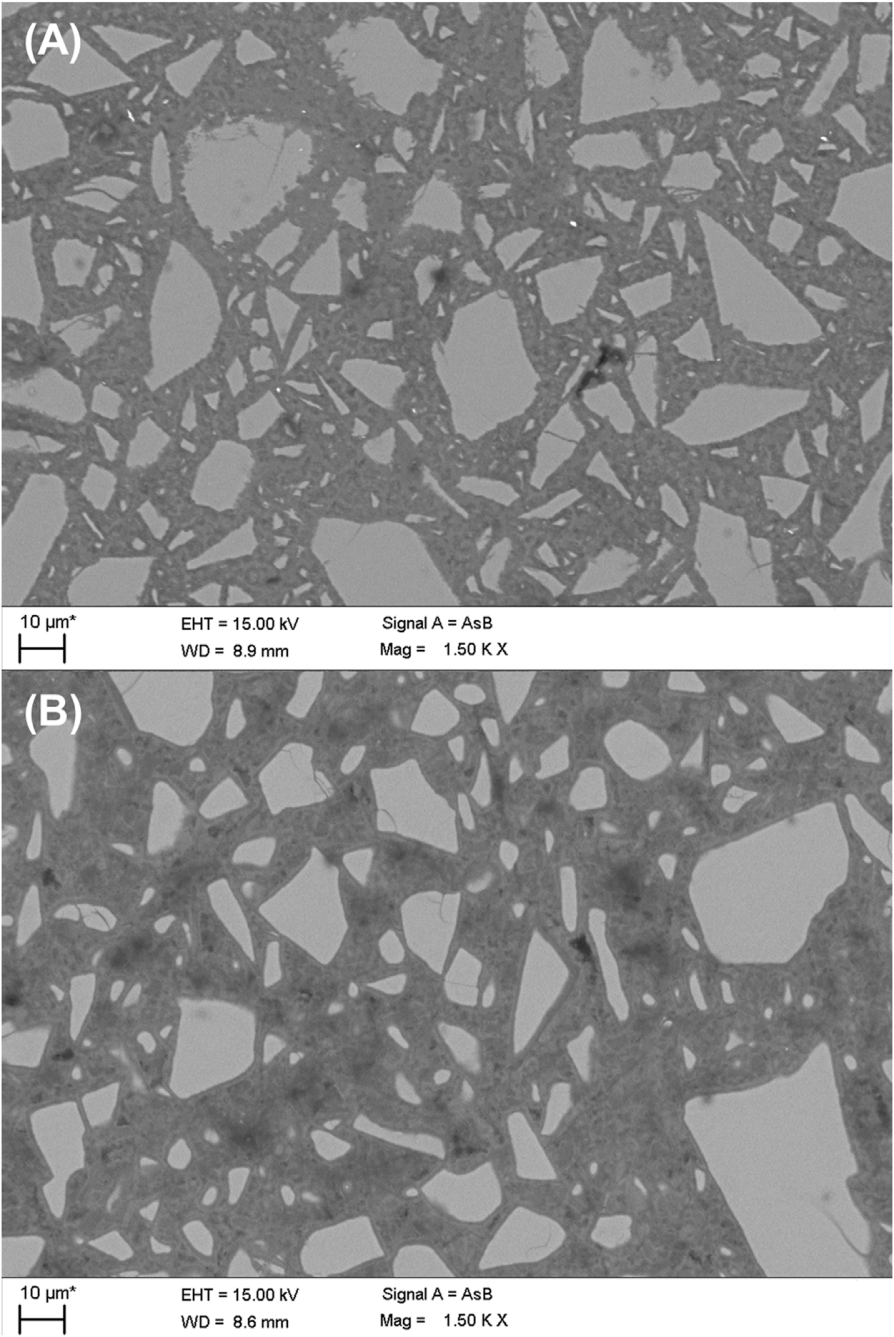
The microstructure of **(A)** alkali-activated BG in comparison to **(B)** AAM from GBFS.

**FIGURE 7:**
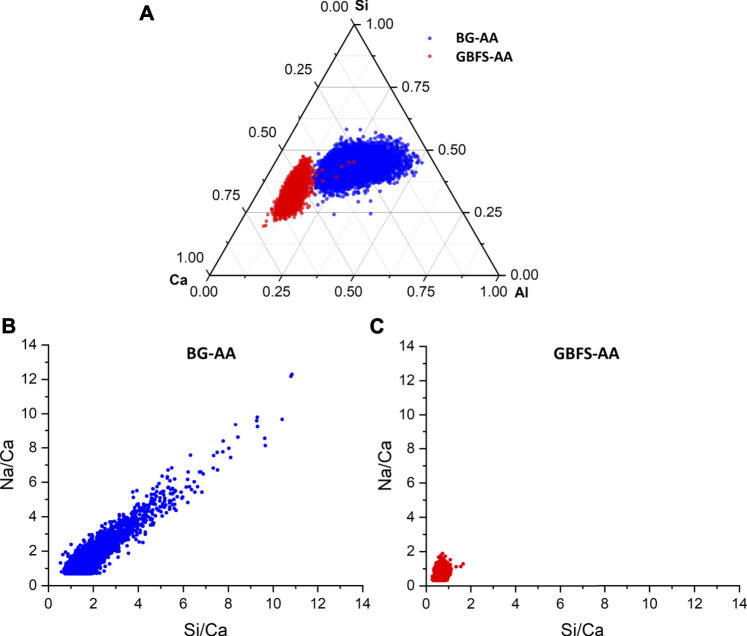
Atomic plots of hydrates in AAMs from BG compared to that of GBFS.

The microstructure for the studied AAMs was investigated by SEM (Figure 6). The alkali-activated BG (Figure 6A) exhibit dense microstructure. In case of GBFS-AA (Figure 6C), the paste attained a relatively compact microstructure with two different C-S-H phases, distinguished by differences in grey scales. The chemical composition, attained from EDS analyses of reaction products in BG-AA compared to that of GBFS-AA, is presented in Figure 7. The result of EDS data points shows different chemistry of hydrates formed in two materials.

### Compressive Strength

**FIGURE 8:**
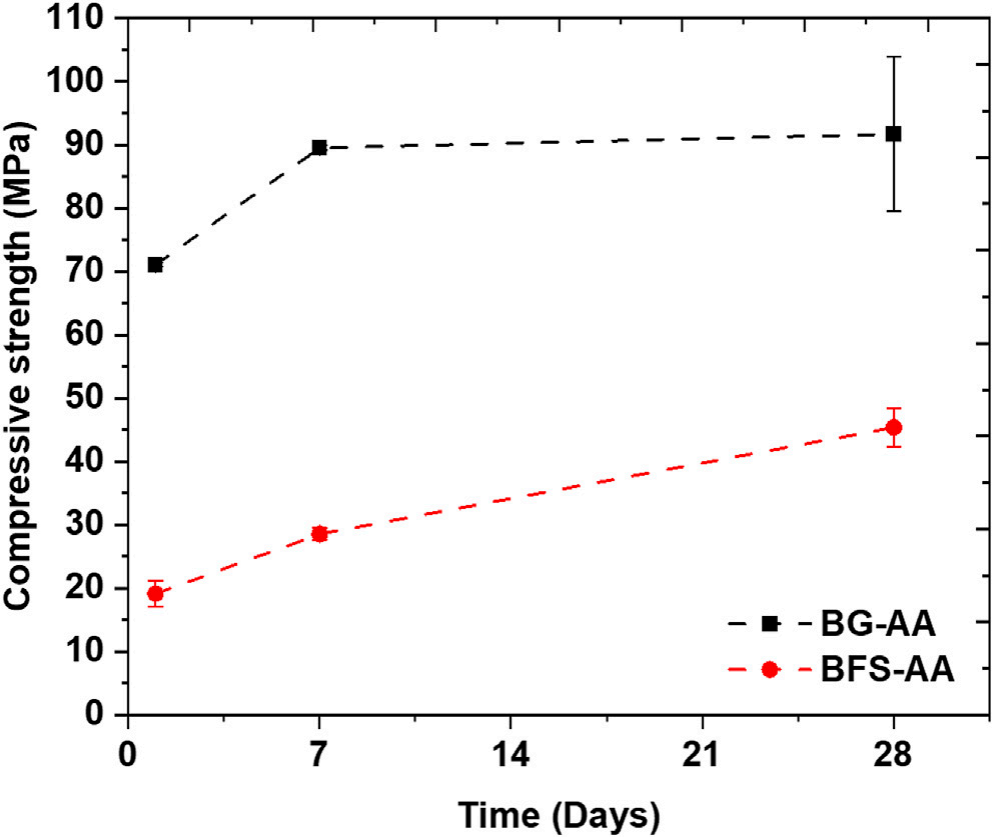
Compressive strength of the prepared AAMs after different periods of hydration.

The compressive strength of the prepared AAMs is presented in Figure 8. BG-AA demonstrated high early compressive strength reaching above 70 MPa after 1d of alkali reaction. In addition, the compressive strength of the BG-AA after different hydration times is higher compared to that of GBFS-AA.

## Discussion

### Hydration Kinetics

In alkali activation reactions the first step is the dissolution of the aluminosilicate source where OH¯ attack the Si-O and Al-O bonds (Yao et al., 2009). This dissolution step is responsible for the first exothermic peak. The decline of this initial peak (Figure 2A) which refers to the slowing down of the wetting and dissolution reaction. The second exothermic peak is ascribed to the continuous dissolution of the aluminosilicates as well as formation of silicate and aluminate oligomers (Yao et al., 2009; Chithiraputhiran and Neithalath, 2013; Sun and Vollpracht, 2018). The third peak is related to polymerization reactions causing the formation of N-A-S-H gel, after which the system reaches a steady stage (Yao et al., 2009). This peak is not apparent in the case of GBFS which indicates that N-A-S-H gel is not among the formed hydration phases in the case of GBFS-AA. The calorimetry results show also that dissolution and polymerization reactions occur at a faster rate for BG-AA compared to GBFS-AA.

### Phase Assemblage

The XRD results (Figure 3) shows that the alkali-activation of BG resulted in the formation of predominantly N-A-S-H in addition to hydrotalcite and C-A-S-H. The amorphous feature has not completely disappeared after the alkali-activation which could be ascribed to the remaining undissolved BG and/or the formation of amorphous products. The alkali reaction products in the case of GBFS were mainly C-A-S-H in addition to hydrotalcite, portlandite (Ca(OH)_2_) and strätlingite. This is due to the high Ca content in the GBFS compared to both BG and MK (Table 1). In addition, N-A-S-H was not detected by XRD in the case of GBFS-AA. The obtained XRD results agree with the calorimetry results in which the exothermic peak associated with the formation of N-A-S-H is only apparent in the case of alkali activation of BG (Figure 2A).

The reaction products were further identified by TGA/DTG (Figure 4). The main weight loss between 50 and 200 C is related to the dehydration of C-S-H related phases such as N-A-S-H and C-A-S-H (Gomez-Zamorano et al., 2017; Humad et al., 2019). The other weight loss (between 200 and 400 C) is ascribed to the decomposition of hydrotalcite-like phases (Humad et al., 2019). These results further confirm the formation of hydrotalcite, as found from XRD analysis (Figure 3).

DRIFT FTIR spectrum of the raw BG glass (Figure 5) display one major peak at 1,170 cm^−1^ which is characteristic to Si-O asymmetric stretching vibration on Q^2^ sites (Yu et al., 1999). Alkali activated BG shows the main Si-O asymmetric stretching peak at about 1,120 cm^−1^ which is in the range of N-A-S-H gel (typically between 1,000–1,100 cm^−1^) (García-Lodeiro et al., 2008). The alkali activation is also associated by the appearance of two peaks at ∼3,400 and 1,650 cm^−1^ which are assigned to the stretching and bending modes of H-O respectively (Guan et al., 2013; Jiang et al., 2018). In addition, the small peak at about 1,400 cm^−1^ is ascribed to the stretching vibration of C-O in carbonates species due to atmospheric carbonation in AAMs (García Lodeiro et al., 2009; Chernyshova et al., 2013; Król et al., 2016). In the case of alkali activated GBFS, the main Si-O stretching vibration was slightly shifted towards lower frequency (∼1,070 cm^−1^). The characteristic Si-O stretching peak of C-S-H typically appears at 970 cm^−1^, but shifts towards higher frequency with increasing Si/Ca (García-Lodeiro et al., 2008). This indicates the formation of C-A-S-H by the alkali activation of GBFS. In addition, the shift towards higher frequency could also be ascribed to the higher degree of polymerization of C-S-H when high alkali concentration is applied (García Lodeiro et al., 2009). In addition, both alkali activated materials show a small peak at about 910 cm^−1^ which is attributed to the Al-O stretching vibration in octahedral Al species (Król et al., 2016). This could further confirm the formation of hydrotalcite as described above. Other small peaks at about 830, 760, and 580 cm^−1^ are attributed to the symmetric stretching vibrations of Si-O, Si-O-Si, and Si-O-Al respectively (Lee and van Deventer, 2003).

The microstructures are distinguishable among alkali-activated mixes (Figure 6). Among the prepared AAMs, BG-AA (Figure 6A) exhibited the densest microstructure. Based on the difference in grey scale, two different C-S-H phases could be distinguished as the hydration products of GBFS (Figure 6B). Haha et al. (2011) reported similar observation in alkali-activated GBFS from NaOH. The two-tone C-S-H (i.e., the layer formed first is brighter than the layer formed later) is due to the differences in nano-porosities and this results in different densities and backscattered electron coefficients (Haha et al., 2011). This is clear in the dark rim surrounding slag grains allocated to later-formed C-S-H. We also observe this two-tone gel in the BG-AA sample. However, this is likely due to the two different precipitates seen in XRD (i.e., N-A-S-H and C-A-S-H gel co-existing in the binder); the Ca-rich gel had brighter grey scale than the N-A-S-H phase. In addition, pores are more visible in BFS-AA samples, similarly observed by Haha et al. (2011), due to the fast formation of dense hydrates from the reaction of the slag and NaOH activator. In contrast, BFS-AA shows much less visible pores in its microstructure. Hydrotalcite did not clearly show up in both samples, although the phase was identified in other characterization techniques. This is due to the high-level intermix in the phase assemblage of both systems.

Regarding the chemical composition of reaction products (Figure 7), the gels in BG-AA had a wide distribution in the Ca content due to the precipitation of both N-A-S-H and C-A-S-H phases (Figure 7A). Notably, the Ca content in these gels is much less than that of GBFS-AA, which is expected based on the chemical composition of the glass compared to the slag. In addition, there was much more Na incorporated in the formation of hydrates in BG-AA in comparison to the GBFS-AA (Figures 7B,C) indicating the formation of the N-A-S-H gel. In contrast, the GBFS-AA formed C-S-H gel with a small fraction Al incorporated while the Ca/Si ratio was ca. 1.6. Furthermore, the hydrates in GBFS-AA did not contain much Na after 7 days. However, after longer hydration times, more Na may incorporate in C-S-H in which the Ca content remains the same, as observed by Haha et al. (2011) in similar binding system. Note that the influences of activators and glass chemistry in the formation of hydrates in BG-AA is of interest for future investigation because these factors were found to strongly influence the reaction of GBFS under alkali activation (Haha et al., 2011, 2011, 2012, 3; Winnefeld et al., 2015).

### Mechanical Properties

The high strength of alkali activated materials indicates high reactivity and dissolution of the applied raw material. In this context, the BG-AA demonstrated ∼70 and ∼90 MPa after 1 and 7 days of hydration respectively. The strength gain for the alkali activated BG is much higher compared to that of alkali activated GBFS which could indicate its higher reactivity and dissolution. Commonly the compressive strength variation for AAMs is not significant after 7 days of reaction (Muñiz-Villarreal et al., 2011). Similar strength was observed after 28 days of hydration (90 ± 15 MPa). It should be noted that the compressive strength for the samples after 28 days of hydration was measured using higher capacity instrument (Zwick/Roell Z100, 100 kN load cell) compared to 1-day and 7-days samples that were tested using Zwick/Roell Z010 (10 kN load cell). However, both measurements were carried out with the same loading rate following BS EN 196–1 standard. This may have resulted in the high standard error value for samples tested at 28 days.

## Conclusion

The aim of the current work was to investigate the structural and mechanical properties of synthetic aluminosilicate glass with basalt composition as a synthetic source for AAMs. The alkali activated glass has demonstrated excellent mechanical properties reaching above 70 MPa after 1 day of reaction. Microstructural analysis showed that the reaction products for the alkali activated glass is composed of N-A-S-H, while C-(A-)S-H is the predominant product for the alkali-activated GBFS. The glass composition is derived from basalt, and thus it could be obtained from naturally occurring silicate minerals with low-raw materials related CO_2_ emission. The results demonstrate the potential of synthetic glass as a new raw material for AAMs. Further work regarding the durability of the developed material as well as investigating alternatives to lower the environmental impact of the melting process is required.

## Data Availability

The original contributions presented in the study are included in the article/supplementary material, further inquiries can be directed to the corresponding authors.
